# Expression profiling in transgenic FVB/N embryonic stem cells overexpressing STAT3

**DOI:** 10.1186/1471-213X-8-57

**Published:** 2008-05-23

**Authors:** Paolo Cinelli, Elisa A Casanova, Syndi Uhlig, Priska Lochmatter, Takahiko Matsuda, Takashi Yokota, Thomas Rülicke, Birgit Ledermann, Kurt Bürki

**Affiliations:** 1 Institute of Laboratory Animal Science, University of Zurich, Winterthurerstrasse 190, CH-8057 Zurich, Switzerland; 2 Department of Stem Cell Regulation, Institute of Medical Science, University of Tokyo, Tokyo 108-8639, Japan; 3 Current Address: Department of Genetics, Harvard Medical School, Boston, MA 02115, USA; 4 Current Address: Institute of Laboratory Animal Science and Research Center Biomodels Austria, University of Veterinary Medicine Vienna, A-1210 Vienna, Austria; 5 Current Address: Novartis Pharma AG, CH-4056 Basel, Switzerland

## Abstract

**Background:**

The transcription factor STAT3 is a downstream target of the LIF signalling cascade. LIF signalling or activation is sufficient to maintain embryonic stem (ES) cells in an undifferentiated and pluripotent state. To further investigate the importance of STAT3 in the establishment of ES cells we have in a first step derived stable pluripotent embryonic stem cells from transgenic FVB mice expressing a conditional tamoxifen dependent STAT3-MER fusion protein. In a second step, STAT3-MER overexpressing cells were used to identify STAT3 pathway-related genes by expression profiling in order to identify new key-players involved in maintenance of pluripotency in ES cells.

**Results:**

Transgenic STAT3-MER blastocysts yielded pluripotent germline-competent ES cells at a high frequency in the absence of LIF when established in tamoxifen-containing medium. Expression profiling of tamoxifen-induced transgenic FVB ES cell lines revealed a set of 26 genes that were markedly up- or down-regulated when compared with wild type cells. The expression of four of the up-regulated genes (Hexokinase II, Lefty2, Pramel7, PP1rs15B) was shown to be restricted to the inner cell mass (ICM) of the blastocysts. These differentially expressed genes represent potential candidates for the maintenance of pluripotency of ES cells. We finally overexpressed two candidate genes, Pem/Rhox5 and Pramel7, in ES cells and demonstrated that their overexpression is sufficient for the maintenance of expression of ES cell markers as well as of the typical morphology of pluripotent ES cells in absence of LIF.

**Conclusion:**

Overexpression of STAT3-MER in the inner cell mass of blastocyst facilitates the establishment of ES cells and induces the upregulation of potential candidate genes involved in the maintenance of pluripotency. Two of them, Pem/Rhox5 and Pramel7, when overexpressed in ES cells are able to maintain the embryonic stem cells in a pluripotent state in a LIF independent manner as STAT3 or Nanog.

## Background

ES cell lines that maintain their pluripotency after transfection and selection procedures are essential for the introduction of selected targeted mutations into the mouse germ-line. Pluripotent ES cells are established *in vitro *from the inner cell mass (ICM) cells of explanted blastocyst-stage embryos [1-3]. Murine ES cells are maintained in a pluripotent state by co-culturing with mitotically-inactivated feeder cells, such as embryonic fibroblasts, and/or the addition of leukaemia inhibitory factor (LIF: [4,5]). These ES cells can be maintained indefinitely in the presence of LIF, and express markers of the undifferentiated and pluripotent state, including the POU-domain transcription factor OCT-3/4 (POU5F1), a factor that is essential for the development of the ICM (reviewed by [6]; [7]). Upon removal of LIF, the cells rapidly lose self-renewal capacity and differentiate into a variety of cell types. LIF belongs to the Interleukin-6 family of cytokines and the members of this family have diverse effects on a variety of cell types [8]. The shared usage of signal transducers (i.e. gp130) in the multichain cytokine receptor complexes clearly explains the functional redundancies of these cytokines (reviewed by [9]).

The pathway by which LIF signalling acts to promote ES cell self-renewal has been partially well studied (reviewed by [10]). LIF signals via heterodimerization of the two class I cytokine receptors, the low affinity LIF receptor (LIFR) and the common subunit, gp130. The cytoplasmic domain of gp130 contains several tyrosinase residues that are phosphorylated by associated JAK (Janus kinase) kinases after ligand-stimulated dimerization. Four of these phosphorylated tyrosines have been identified as putative interaction sites with the SH2 (Src homology 2) domain of the transcription factor STAT3 (signal transducer and activator of transcription; [11]). Stimulation of gp130 signalling in ES cells also phosphorylates SHP-2 (SH2-domain-containing tyrosine phosphatase) and leads to activation of the mitogen-activated protein (MAP) kinases ERK1 and ERK2 [12]. Inhibition of the SHP-2/RAS/ERK pathway promotes self-renewal and suppresses differentiation and treatment of mouse ES cells with the MAPK-inhibitor PD098059 [13] was shown to enhance self-renewal [14].

Matsuda et al. (1999) have shown that activation of the STAT3 transcription factor is sufficient to maintain mouse ES cells in an undifferentiated state in the absence of LIF [15]: An inducible transgene construct encoding the entire STAT3 coding region fused to the mutated ligand-binding domain of the estrogen receptor (STAT3-MER) was introduced into ES cells. ES cells expressing the STAT3-MER fusion protein maintained their undifferentiated state in the presence of OHT and in the absence of LIF [15]. This study highlighted the importance of STAT3 pathway in maintenance of ES cell pluripotency *in vitro*. However, the *in vivo *relevance of the LIF pathway is to date still not clear; LIF expression can be detected in the trophectoderm (TE) of the blastocyst whereas LIF receptor is expressed in the ICM. However, neither LIF mutants [16] nor mutants of the receptors LIFR [17,18] and gp130 [19] result in any defects in the development of the ICM or early epiblast. Recent evidence suggests that the LIF pathway is necessary for survival of the mouse epiblast during diapause [20].

ES cell lines derived from different mouse strains exhibit variable degrees of LIF dependency as demonstrated in STAT3 gene targeting experiments by Raz et al. [21]. ES cells heterozygous for a STAT3 mutation could only be established from E14 cells (129P2/OlaHsd; [22]). Targeted clones from other cell lines were invariably trisomic for chromosome 11 that carries the STAT3 locus, and thus retained normal levels of activated STAT3.

To date it is unclear if higher amounts of STAT3 in the inner cell mass of blastocyst support the survival and derivation of pluripotent ES cells, especially in so called non-permissive mouse strains like FVB/N. The inbred mouse strain FVB/N is widely used for the generation of transgenic animals [23], however only one germline competent ES cell line has been reported [24]. We therefore, in a first step, generated FVB/N transgenic mice overexpressing a tamoxifen inducible STAT3 (STAT3-MER). Our data demonstrated that overexpression of STAT3 in the ICM of the blastocyst supports the establishment of ES cells in the FVB/N mouse strain. ES cell lines overexpressing STAT3-MER were germline-competent whereas the only WT line that we could establish was not germline-competent.

Recent studies have begun to identify key players involved in the intracellular signal transduction pathways regulating stem cell renewal and proliferation. Several transcription factors including the OCT-3/4 have been shown to be essential to maintain pluripotency in the ICM, but none had been shown to function independently of the LIF pathway with exception of the newly identified homeobox transcription factor Nanog, that directs pluripotency in mouse ICM and mouse ES cells and functions independently from LIF dependent STAT3 activation [25,26]. Nanog is detected in the ICM and early germ cells, as well as in the ES and embryonic carcinoma (EC) cell lines derived from these stages [25]. Overexpression of Nanog relieves mouse ES cells cultured without feeder cells in the presence of serum from dependence on LIF stimulation for self-renewal whereas Nanog-deficient mouse ES cells loose pluripotency and differentiate into extra embryonic endoderm lineages [26].

We have further focused our study on the STAT3 pathway in order to elucidate the differences between WT and STAT3 overexpressing embryonic stem cells. We performed microarray-analysis comparing WT and STAT3-MER overexpressing FVB/N ES cells and identified a pool of genes that were differentially expressed. From the microarray dataset, we screened for potential candidates of pluripotency by their expression pattern in the early preimplantation embryo. Among these, we confirmed Pem/Rhox5 and Pramel7 as regulators of pluripotency using functional studies: ES cells overexpressing Pem/Rhox5 and Pramel7 were able to maintain typical pluripotent ES cell morphology in the absence of LIF, as well as the characteristic pluripotency-related markers SSEA-1 and Oct4 in a similar extent as Nanog which was used as a positive control. This clearly demonstrates that these two STAT3-pathway related genes are involved in the maintenance of pluripotency.

## Results

### Generation of transgenic mice overexpressing STAT3-MER

We produced FVB/N transgenic mice overexpressing a fusion protein composed of the entire coding region of mouse STAT3 and the modified ligand-binding domain (G525R) of the mouse estrogen receptor [15]. The modified ligand-binding domain binds the synthetic steroid ligand 4-hydroxytamoxifen (4OHT) but not 17β-estradiol [30]. The expression of the transgene is driven by a chicken β-actin promoter and therefore is expected to be ubiquitously expressed. After injection of the construct into the pronucleus of fertilized FVB/N eggs we obtained seven positive founder animals. When crossed with wild-type FVB/N partners, six of them showed germline transmission. A multiple tissue analysis was performed, by western blotting, in order to define animals exhibiting ubiquitous expression (data not shown). Two of highest STAT3-MER expressing lines (Tg741 and Tg743) were selected for further experiments. Both transgenic lines contained a single integration of the transgenic cluster and were maintained in a hemizygous state by constant breeding with WT FVB/N mice.

### Overexpression of inducible active STAT3-MER enables the establishment of germline competent ES cells from FVB/N blastocysts

**Figure 1:**
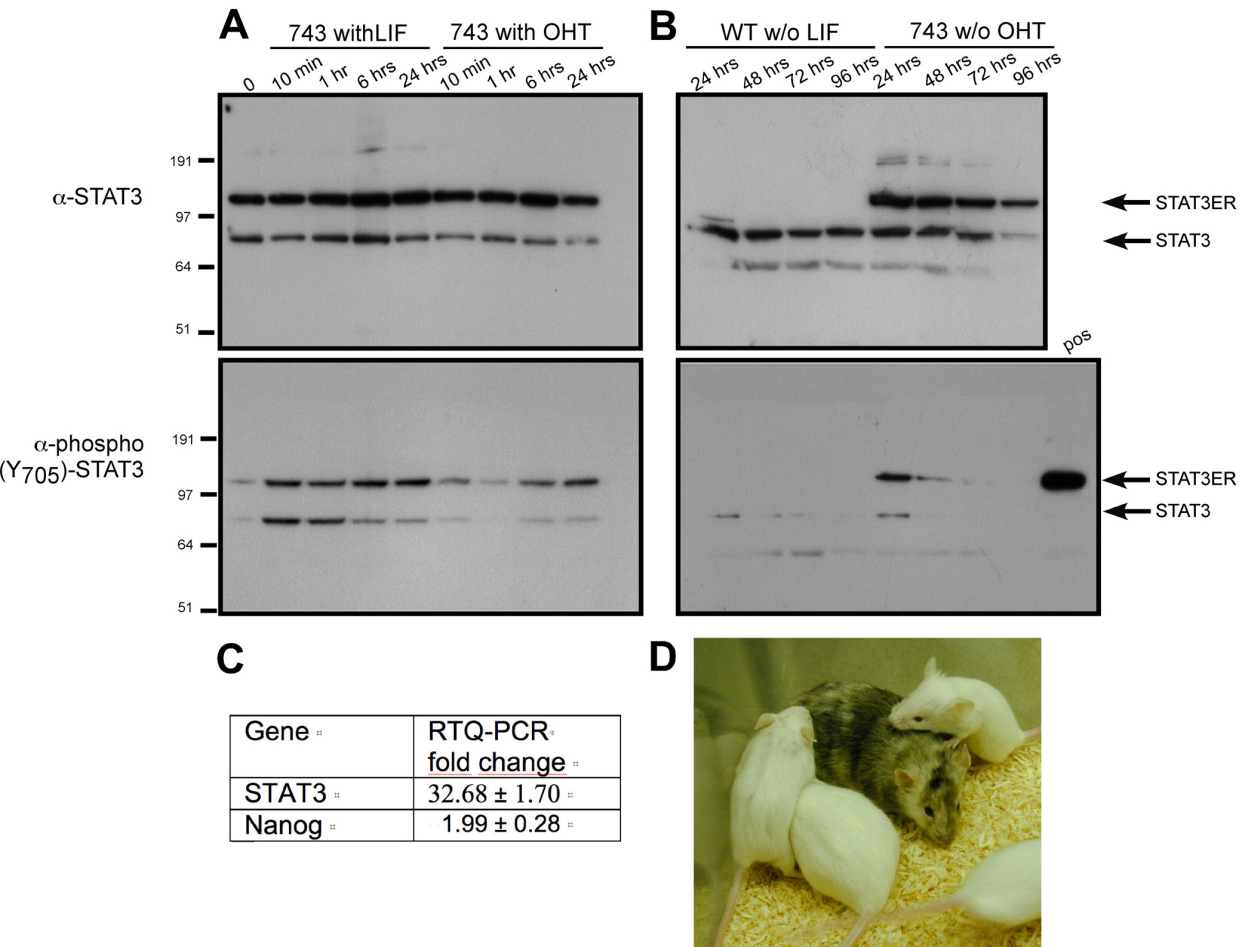
**Characterization of the 743 transgenic ES cell line**. **A. **Phosphorylation/dephosphorylation analysis of Tyr705 in endogenous and transgenic STAT3. After 24 hours of LIF and OHT deprivation cells were cultivated till their homogenization in presence of either LIF or OHT. During LIF or OHT deprivation no changes in the protein expression levels could be detected, but after 24 hrs the Tyr705 residue of both transgenic and WT STAT3 was completely dephosphorylated. 10 minutes after addition of LIF the tyr705 residue of both STAT3 was phosphorylated whereas after addition of OHT complete phosphorylation was obtained only after 6 hrs. **B. **Dephosphorylation of Tyr705 was analyzed by eliminating LIF or OHT from respectively WT or 743 cells. Kinetics for the dephosphorylation were slower then for the phosphorylation, only after 48 hrs dephosphorylation of WT Tyr705 was complete whereas complete dephosphorylation of STAT3-MER occurred only after 72 hrs. **C. **Nanog and STAT3 expression levels were tested by RTQ-PCR. Values are normalized with β-actin and the values indicate fold changes compared to the WT. Both WT and 743 ES cell lines expressed Nanog and the transgenic 743 ES cells have an increased Nanog expression compared to WT. **D.** After injection of 743 in C57BL/6 host blastocysts a 50–60% chimera was generated. Littermates from the crossing of the chimera with a WT FVB/N female generated white littermates, 50% of which were hemizygous for the transgene, indicating germline competence of the 743 cell line.

In order to test if the overexpression of STAT3-MER would allow the establishment of germline competent ES cells from the FVB/N mouse strain, morulae were flushed from the uterotubal junction 3 days after mating and cultured overnight in M16 medium (Sigma). Fully expanded blastocysts were transferred onto MEF as described in "Methods". Full-grown ICMs were picked from the outgrown TE and dissociated mechanically into groups of cells and these aggregates reseeded onto embryonic feeder fibroblasts. 3–4 days later, compact stem cell colonies could be identified. Single colonies were dissociated as described above and reseeded. Non-differentiating clonal lines were further passaged and split after 2–3 generations for further characterization. Embryos were cultivated in medium containing either LIF or OHT. We were able to establish both wild type and transgenic ES cell lines. However, even if ICMs from WT embryos were able to outgrowth from the TE in presence of OHT it was impossible to generate ES cell colonies during the further steps of cultivation and WT ES cells were only obtained when LIF was present in the medium (Table 1). When using OHT-supplemented medium without LIF 43–71% of the embryos from both Tg741 and Tg743 transgenic lines yielded ES cell lines, all of which were transgenic (Table 1). Because the mice used were hemizygous for the transgene, and therefore only 50% of the embryos is expected to be transgenic, it is fair to assume that we were able to derive ES cells from virtually all the transgenic embryos. These results strongly indicate a supportive effect of active STAT3-MER on the maintenance of pluripotent ES cells. The newly established ES cell lines overexpressing STAT3-MER were cultivated further only in presence of OHT without LIF. In order to confirm the pluripotency of these ES cells, karyotypically normal male cells from both transgenic lines (Tg741 and Tg743) were injected into C57BL/6 host blastocysts. Chimeric males were identified by the absence of eye (pink) and coat (albino) pigmentation and mated to wildtype FVB/N females. Germline transmission of the FVB/N ES cell genome resulted in albino offspring (Figure 1D). None of the 5 FVB WT ES cell lines was able to produce chimeras when injected into C57BL/6 blastocysts (data not shown). To confirm that overexpression of active STAT3 supports the survival and derivation of pluripotent ES cells also in the F1 generation, transgenic germline F1 offspring from the line Tg741 were mated to wildtype animals. Blastocyst stage embryos were isolated and cultivated as previously described, if cultivated in presence of OHT stem cell lines could be established from 44% of the embryos, all lines being transgenic (Table 1).

**Table 1:** Establishment of FVB/N ES cells

**Founder Line**	**No Of embryos**	**Medium Supplements**	**No of ICM picked**	**No of stem cell colonies**	**Cell lines**	**Transgenic**
WT	15	LIF	8 (53%)*	5 (62%)**	5	-
	15	OHT	4 (27%)*	0 (0%)**	-	-
741	28	OHT	23 (82%)*	10 (43 %)**	7	7 (100 %)^#^
743	28	OHT	14 (50%)*	10 (71 %)**	10	10 (100 %)^#^
741-F1	26	OHT	18 (69%)*	8 (44 %)**	8	8 (100 %)^#^

* percent ICM per plated embryo; ** percent colonies per picked ICM

^# ^percent transgenic per cell lines

WT FVB/N or hemizygous FVB/N males carrying the STAT3-MER transgene (lines 741 and 743) were mated with WT FVB/N females. Blastocysts were cultivated either in presence of LIF or OHT. Outgrown ICM's were picked and cultivated till ES cell colonies were visible. Cells were tested for the presence of the transgene by PCR analysis. As expected it was possible to establish WT ES cells only in presence of LIF but not when OHT alone was added to the culture. Furthermore in presence of OHT both transgenic lines 741 and 743 generated ES cell colonies that were carrying the transgene. Because theoretically only 50% of the blastocysts were expected to be transgene it is to assume that the establishment frequency was almost 100%. After generation of germline competent chimera with the newly established 741 cell line hemizygous transgenic F1 animals were generated and mated with WT FVB/N females. In this breeding the establishment efficiency in presence of OHT was similar to one observed in the parental blastocysts indicating that the STAT3-MER induced stabilization is kept also in the F1 generation.

### Characterization of the newly established FVB/N ES cells overexpressing STAT3-MER

The expression level of STAT3-MER in the ES clones obtained from the line 743, was tested by western blot (see Figure 1A and 1B). Upon LIF stimulation STAT3 is phosphorylated on the tyrosine residue (Y705), dimerizes and can bind DNA [31,32]. In order to test if OHT is able to induce STAT3-MER phosphorylation FVB/N ES cells expressing STAT3-MER were first deprived of LIF or OHT for 24 hrs, after this time the Tyr705 residues of both the endogenous STAT3 and STAT3-MER were completely dephosphorylated. After the 24 hrs deprivation cells were stimulated either with LIF or OHT for 10 minutes up to 24 hours and further cultivated in presence of LIF or OHT till their homogenization. Cell extracts were separated by SDS-PAGE, blotted and probed with anti-STAT3 and anti-phospho (Y705) antibodies. LIF stimulation induced tyrosine phosphorylation of both endogenous STAT3 and STAT3-MER (Figure 1A). As previously observed [15], endogenous STAT3 was rapidly phosphorylated whereas phosphorylation kinetics of STAT3-MER were slower. In ES cells derived from Tg743 stimulation with OHT resulted in a strong tyrosine phosphorylation of STAT3-MER, but only a limited phosphorylation could be detected for endogenous STAT3 (Figure 1A). During the 24 hours of induction with either LIF or OHT expression of Oct4 was confirmed (data not shown). Dephosphorylation kinetic of Tyr705 was also analyzed by eliminating LIF or OHT from the medium of respectively WT or 743 cells. Kinetics for the dephosphorylation were slower then for the phosphorylation, only after 48 hrs dephosphorylation of WT Tyr705 was complete whereas complete dephosphorylation of STAT3-MER occurred only after 72 hrs. Dose dependence for dephosphorylation could also be observed. In ES cells derived from the Tg743 line, expressing higher amounts of STAT3-MER, dephosphorylation was slower compared to cells derived from the lower expressing Tg747 line (data not shown).

### ES cells overexpressing STAT3-MER express the typical ES-cell markers

**Figure 2:**
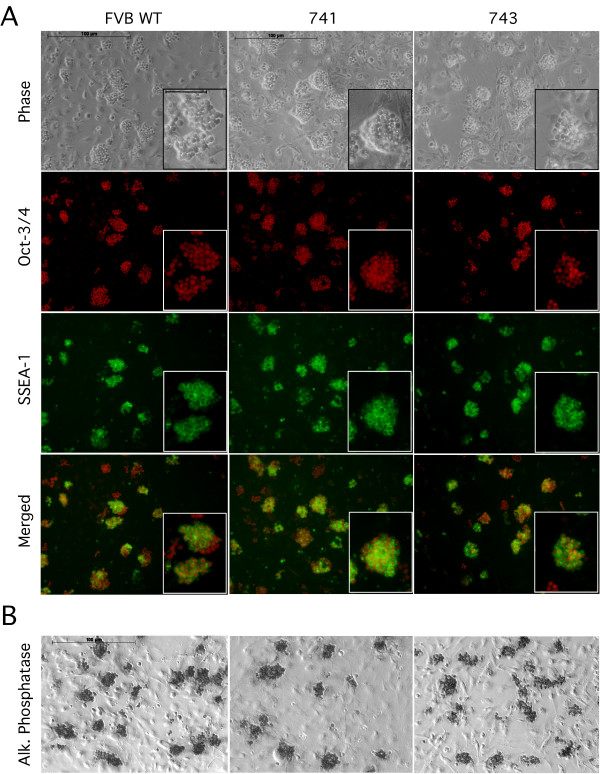
**Immunohistochemical analysis of WT, 741 and 743 ES cell lines**. All three cell lines express the nuclear marker OCT-3/4 and the surface marker SSEA-1 (A) and alkaline phosphatase (B). The expression of all markers was restricted to ES cells. Mouse fibroblast used as feeder cells are negative for OCT-3/4, SSEA-1 and alkaline phosphatase. Scale bar in both large and small panels: 100 μm.

ES cells, as well as cells of the ICM of mouse blastocysts, express a panel of markers that are used to characterize undifferentiated, pluripotent embryonic cells, between them Nanog, alkaline phosphatase, OCT-3/4 and SSEA-1 are the most typically used. Nanog expression was tested by RTQ-PCR; both WT and 743 ES cells expressed Nanog and a light overexpression could be detected in the 743 cells if compared with WT cells after normalization with the housekeeping gene β-actin (Figure 1C). The expression of the transcriptional factor OCT-3/4 and the surface marker SSEA-1 was tested by immunohistochemistry (Figure 2A); WT FVB as well as both transgenic lines 743 and 741 expressed both markers. Furthermore, all three cell lines expressed the marker alkaline phosphatase (Figure 2B). In all cases the expression was restricted to the ES cells and no signal could be detected in the inactivated fibroblast used as feeder cells.

### Microarray analysis

Even though it was possible to establish WT FVB ES cells in presence of LIF and these cells express the typical markers for ES cells they were not able to generate chimeric mice. This suggests that overexpression of STAT3-MER could increase the level of pluripotency in FVB ES cells. In order to understand the difference between the WT cells and the germline competent 743 cells we decided to compare the gene expression profiles of both lines. We compared three independently cultivated dishes of WT FVB cells cultivated in the presence of LIF with three independently cultivated dishes of the transgenic 743 cells overexpressing STAT3-MER cultivated in the presence of OHT. Total RNA was isolated and an expression analysis was performed by hybridizing U74v2 Affymetrix chips containing probes covering the complete mouse transcriptome (36'000 transcripts). Analysis was performed with dCHIP by using both the PM/MM (perfect match/mismatch) difference model and the PM (perfect match) only model in order to compare the results [33]. Genes showing expression changes higher then 1.5 fold were considered. As control, we first confirmed that the overexpression of STAT3-MER in the 743 line was 33 times higher than in the WT cells. We further identified a set of 26 differentially regulated genes, 13 were upregulated (Table 2) whereas 13 were downregulated (Table 3).

**Table 2:** Upregulated genes

**Probe set**	**Identifier**	**RefSeq**	**Chr**	**MM/PM fold change**	**MM/PM p-value (t-test)**	**PM fold change**	**PM p-value (t-test)**	**RTQ-PCR fold change**
98524_f_at	**X-linked myotubular myopathy gene 1 (Mtm1)**	NM_019926	X	1.87	0.018906	1.91	0.017234	n.d.
94375_at	**Hexokinase 2 (Hk2)**	NM_013820	6	1.6	0.018958	1.61	0.015899	1.79 ± 0.11
95545_at	**Insulin-like growth factor 1A (Igf1)**	NM_010512	10	2.34	0.003079	1.6	0.007358	n.d.
98059_s_at	**lamin A (Lmna)**	NM_001002011	3	1.58	0.070634	1.47	0.035881	n.d.
95562_at	**Nuclear domain 10 protein 52 (Ndp52)**	NM_029755	11	2.66	0.065377	2.08	0.057843	6.72 ± 0.67
101368_at	**Reproductive homeobox 5 (Rhox5/Pem)**	NM_008818	X	1.68	0.029649	1.82	0.013529	3.64 ± 0.3
103470_at	**Pramel6**	NM_178249	2	6.18	0.02356	4.14	0.027034	10.0 ± 0.37
93245_at	**Pramel7**	NM_178250	2	3.51	0.051948	2.77	0.052927	8.51 ± 0.92
95609_at	**Protein phosphatase 1, regulatory (inhibitor) subunit 15b (Ppp1r15b)**	NM_133819	1	1.6	0.024109	1.5	0.022545	1.56 ± 0.12
92232_at	**suppressor of cytokine signaling 3 (Socs3)**	NM_007707	11	1.75	0.053978	1.56	0.015837	2.34 ± 0.35
101449_at	**tripartite motif-containing 41 (Trim41)**	NM_145377	11	-	-	4.11	0.000819	1.07 ± 0.17
160365_at	**Eukaryotic translation initiation factor 2, subunit 2 beta (Eif2s2)**	NM_026030	2	1.98	0.040383	1.96	0.042002	1.80 ± 0.09
160420_r_at	**tubulin, alpha 3A (Tuba3a)**	NM_009446	6	2.07	0.080906	1.93	0.078476	n.d.

Genes upregulated after STAT3-MER activation through OHT. Microarray values are given as fold changes ratios relative to normalization derived from dCHIP. 13 genes were found to be significantly upregulated when compared with WT FVB/N cells cultivated in presence of LIF. Only genes with a 1.5× fold increase were considered as significant. The mRNA expression levels (mean ± standard deviation, n = 3) of relative to FVB WT control cells were validated by real time PCR analysis, RTQ-PCR values are ratios relative to β-actin (housekeeper gene) expression. For all selected genes measured, RT-PCR corroborated the rank order of magnitude of expression measured on the microarrays. Calculations were performed with both the PM/MM (perfect match/mismatch) difference model and the PM (perfect match) only model in order to compare the results.

**Table 3:** Downregulated genes

**Probe set**	**Identifier**	**RefSeq**	**Chr**	**MM/PM fold change**	**MM/PM p-value (t-test)**	**PM fold change**	**PM p-value (t-test)**	**RTQ-PCR fold change**
97283_at	**developmental pluripotency-associated 3 (Dppa3)**	NM_139218	6	-2.16	0.10982	-2.81	0.047749	-11.4 ± 1.51
133948_at	**Eukaryotic translation initiation factor 2C 4 (Eif2c4**)	AI504948	17	-1.53	0.044727	-1.46	0.018363	n.d.
134566_at	**Left-right determination factor 2 (Lefty2)**	NM_177099	1	-2.7	0.029827	-2.32	0.025196	-2.35 ± 0.23
103389_at	**aminoadipate-semialdehyde synthase (Aass)**	NM_013930	6	-2.07	0.077262	-1.71	0.100000	n.d.
167617_r_at	**transmembrane protein 109 (Tmem 109)**	NM_134142	19	-1.89	0.036619	-1.73	0.042999	n.d.
166131_at	**enabled homolog (Enah)**	NM_001083120	1	-1.55	0.008738	-1.46	0.016412	n.d.
138014_at	**nucleoporin 153 (Nup 153)**	NM_175749	13	-1.55	0.012282	-1.49	0.011324	n.d.
167824_f_at	**S-phase kinase-associated protein 2 (p45/Skp2)**	NM_013787	15	-1.74	0.054672	-1.62	0.073491	-1.27 ± 0.03
100499_at	**syntaxin 3 (Stx3)**	NM_001025307	19	-2.09	0.033994	-2.03	0.036655	n.d.
166600_at	**KH domain containing, RNA binding, signal transduction associated 3 (Khdrbs3)**	NM_010158	15	-1.66	0.027322	-1.52	0.035974	n.d.
93568_i_at 93569_f_at	**RIKEN cDNA 2610042L04 gene**	BC096548.1	14	-3.29	0.069938	-2.94	0.070848	n.d.
161004_at	**RIKEN cDNA 1700097N02 gene**	XM_001479022	17	-2.12	0.100000	-2.24	0.002726	n.d.
167843_f_at	**similar to RIKEN cDNA 1110051B16 gene**	XM_001472554	14	-2.71	0.004646	-1.81	0.003623	n.d.

Genes downregulated after STAT3-MER activation through OHT. 13 genes were found to be significantly downregulated when compared with WT FVB/N cells cultivated in presence of LIF. Only genes with a 1.5× fold decrease were considered as significant. The expression levels of selected genes were confirmed by real time PCR analysis. Calculations were performed with both the PM/MM (perfect match/mismatch) difference model and the PM (perfect match) only model in order to compare the results.

### In situ Hybridization

**Figure 3:**
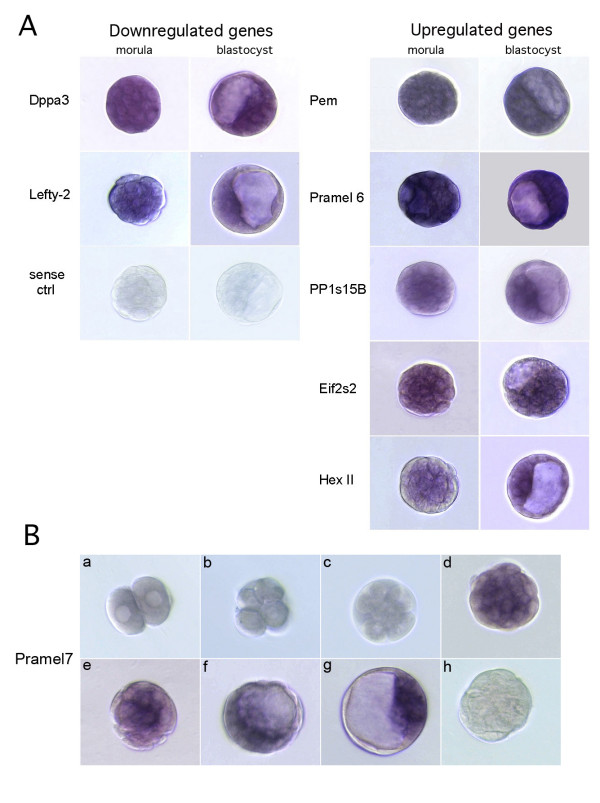
***In situ *hybridization of the eight selected genes**. **A. **Dppa3, Eif2s2, Pem/Rhox5 and Pramel6 exhibited a general expression in all cells of the morula and of the blastocyst. Expression of Hexokinase II, Lefty2 and PP1s15B was restricted to the cells of the inner part of the morula and to the ICM in the blastocyst. Sense probes in the same concentration of the antisense probes were used as negative controls for the hybridization. **B. **In situ hybridization with a Pramel7 antisense riboprobe of preimplantation embryos (a: two-cell embryo, b: four-cell embryo, c: eight-cell embryo; d, e: compacted morula, f, g: blastocyst, h: negative control with sense riboprobe). Magnification 40×.

In a first step we analyzed which of the differentially expressed genes had already previously been described in the literature to be expressed during preimplantation mouse development and therefore potentially play a role in maintenance of pluripotency. Eight genes out of the 26 identified were considered as candidates to have a potential function in determination and maintenance of pluripotency in ES cells. For these genes *in situ *hybridization was performed in order to define the regions of preimplantation embryos in which they were expressed. The temporo-spatial expression was analyzed by whole mount *in situ *hybridization of morulae and blastocysts. Four genes, Pramel7, Lefty2, Protein Phosphatase 1 regulatory subunit 15B and hexokinase II were expressed only in the central part of the morula and in the ICM of the blastocyst (Figure 3A). The other five genes, Pramel6, Eif2s2, Pem/Rhox5, Dppa3 and Skp2 were found to be expressed in all cells of the morula and blastocysts (Figure 3A). Because the Pramel7 expression was restricted to the central part of the morula and in the ICM of the blastocyst, a more exact analysis of the preimplantation stages was performed (Figure 3B). Expression of Pramel7 starts at the compacted morula stage, no expression could be detected in earlier developmental stages indicating that this gene fulfils the requirements for being a potential candidate involved in maintenance of pluripotency. A similar expression pattern can be observed for Nanog [25].

### Overexpression of Pem/Rhox5 and Pramel7 is sufficient for maintenance of ES cells in the absence of LIF

**Figure 4:**
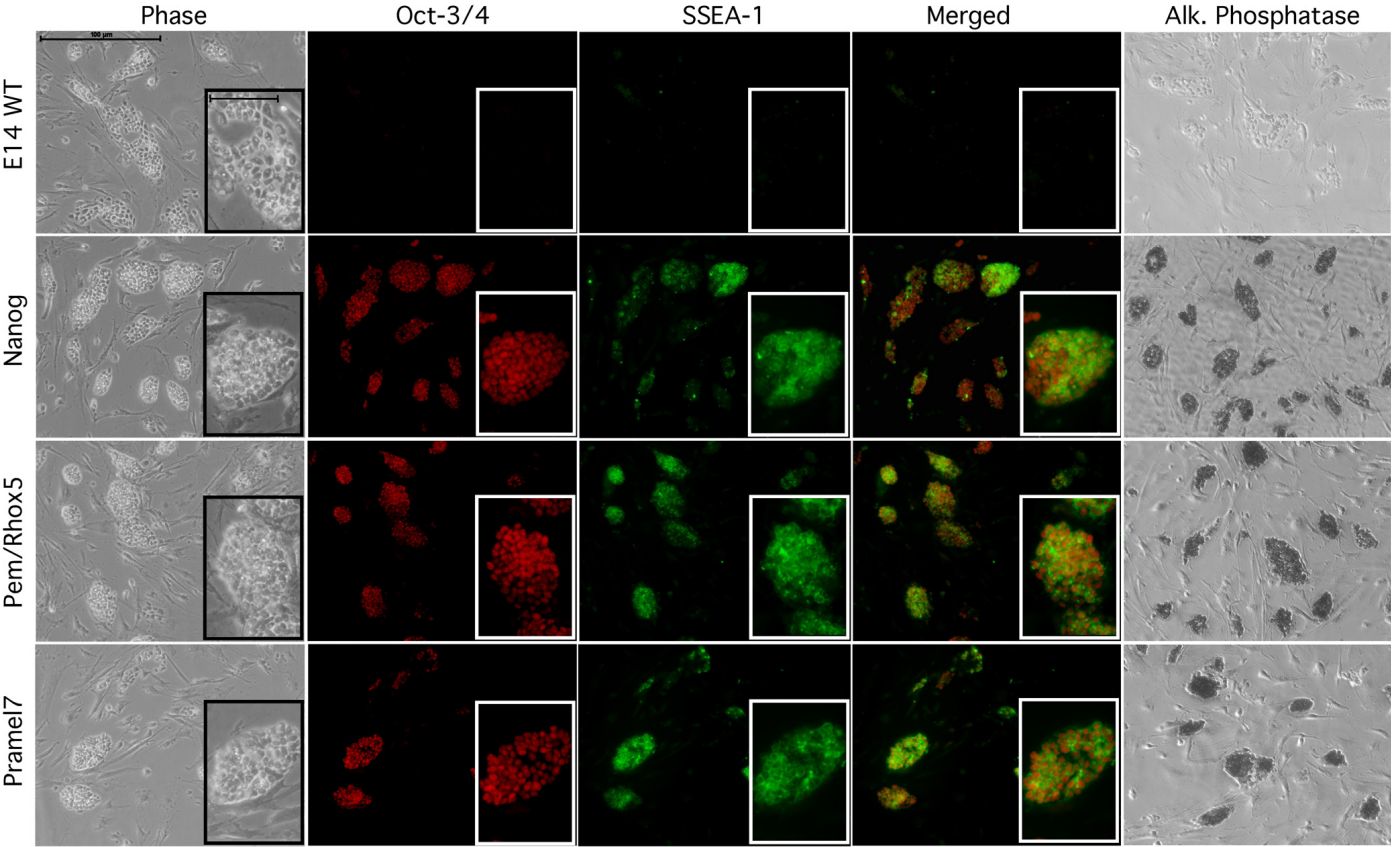
**Immunohistochemical analysis of WT E14 ES cells and E14 overexpressing, Nanog, Pem and Pramel7**. IHC analysis with OCT-3/4 and SSEA-1 and staining for alkaline phosphatase were performed after cultivation of the cells for 8 days without adding LIF to the medium. E14 ES cells differentiated under these conditions and lost the expression of OCT-3/4 and SSEA-1 and alkaline phosphatase. Both Pem/Rhox5 and Pramel7 overexpressing cells maintained the expression of the pluripotency markers. Nanog overexpressing cells, used as a control for the experiment, as expected maintained their pluripotency. Scale bar in both large and small panels: 100 μm.

In order to test if Pramel7 is able to maintain pluripotency without direct activation of the STAT3 cascade through LIF the full-length cDNA of Pramel7 was inserted in the pfloxedNanog vector (see Chambers et al., 2003) instead of the cDNA of Nanog, and the vector was electroporated in E14 ES cells. In parallel the full-length cDNA of Pem/Rhox5 was also cloned in the same way into the pfloxedNanog vector. Pem/Rhox5 was previously described to play a role in maintenance of pluripotency, but it is not yet known if it is transcriptionally regulated through STAT3. As a control for the experiments the pfloxedNanog vector itself was also electroporated in E14 cells. All electroporated cells were selected with puromycin and resistant colonies were picked and expanded. After testing for the presence of the vectors by PCR the positive clones were analyzed by real time PCR and the clones with the strongest expression were used for further experiments. In order to test for the capacity of maintaining pluripotency in absence of LIF, the cells were cultivated for 8 days without addition of LIF to the medium. After 8 days in culture IHC was performed in order to detect the expression of OCT-3/4, SSEA-1 and alkaline phosphatase (Figure 4). E14 WT ES cells started after 4 days to differentiate and showed the typical flattened morphology of differentiating cells (data not shown), after 8 days the cells were completely differentiated and no longer expressed OCT-3/4 and SSEA-1. Nanog overexpressing cells as expected maintained their pluripotent state also in absence of LIF. Both Pramel7 and Pem/Rhox5 overexpressing clones showed a similar behaviour as Nanog overexpressing cells. The colonies maintained the typical round shaped morphology and expression of OCT-3/4 and SSEA-1 was present indicating that these two genes were able to maintain pluripotency also in absence of LIF.

## Discussion

ES cell lines derived from different mouse strains exhibit variable degrees of LIF dependency and to date it was unclear if higher amounts of STAT3 in the inner cell mass of blastocyst could support the survival and derivation of pluripotent ES cells in non-permissive mouse strains. Our work indicates that the activation of the STAT3 pathway during cultivation of blastocysts supports ICM outgrowth and clearly favors the establishment of new ES cell colonies in the so called non-permissive FVB mouse strain.

Even though we were able to establish WT FVB/N ES cells in the presence of LIF these cells were not fully pluripotent and were unable to generate chimeras. Only through the overexpression of STAT3-MER cells we were able to establish germline competent FVB/N ES cells. Furthermore, in presence of OHT both transgenic lines 741 and 743 generated ES cell colonies with a very high efficiency. Theoretically only 50% of the blastocysts were expected to be transgenic and this is also the establishment efficiency we obtained. It is therefore fair to assume that the establishment frequency was almost 100% (Table 1).

Interestingly no changes in the regulation of the classical marker for pluripotent ES cells could be detected between WT and 743 cells. Alkaline phosphatase, OCT-3/4, SSEA-1 were correctly expressed in both wildtype and transgenic ES cell lines (see Figure 2A and 2B). RTQ-PCR showed a slight upregulation of Nanog in the 743 ES cells (Figure 1C). If this upregulation is due to a direct or an indirect interaction with STAT3 has to be further analyzed. The different roles of the LIF pathways and of Nanog are yet not very clear. ES cell populations are heterogeneous and it is also known that Nanog is expressed discontinuously in pluripotent cells in the embryo and it is therefore to assume that Nanog and the LIF pathway interact to some extent in controlling the different events that regulate pluripotency and self-renewal. The recent findings of Chambers et al. (2007) corroborate this hypothesis. The authors demonstrated that Nanog expression was specifically required for both the formation of the ICM and of the germ cells, rather than for the housekeeping machinery of pluripotency embryonic, and that stem cells could self-renew indefinitely in the permanent absence of Nanog [34].

We were further interested in the identification of the molecular changes induced through STAT3-MER overexpression. We therefore decided to identify STAT3-pathway related genes by expression profiling. In general, there is great interest in identifying the signature of stemness by the constellation of genes that stem cells express. DNA microarray technology allows the discovery of a large number of genes that are thought to be the molecular signature of mouse ES cells. Recently, global transcription profiles of undifferentiated ES cells and blastocyst have been reported by several groups, most of these studies were comparing differentiated versus undifferentiated cells [35-38]. In our study the FVB/N WT cells and the FVB/N cells overexpressing STAT3-MER are very similar, they just express different amounts of the transcription factor STAT3 and therefore we assumed that they would differentially regulate only a few genes. We compared gene expression changes between FVB/N ES cells overexpressing activated STAT3 cultivated in the presence of OHT and the absence of LIF and WT FVB cells cultivated in the presence of LIF by microarray analysis and identified a group of 26 genes that showed significant differential expression. From this list we preselected interesting genes by a careful literature and gene-expression databank analysis and identified which genes were characteristically expressed during the mouse preimplantation development.

These genes can be attributed to different categories according to their function: The first group contains regulatory members of the STAT3 pathway that are involved in the regulation of downstream events of the JAK/STAT cascade (like SOCS-3, protein phosphatase-1 regulatory subunit 15B and Eif2s2), the second group of genes is involved in the regulation of ES cell metabolism, whereas the third group contains genes that are involved in pluripotency maintenance and cell viability.

In the first group, among others, we found upregulation of SOCS-3 in STAT3-MER overexpressing cells. SOCS3 is a member of the suppressor of cytokine signalling (SOCS) family which has been implicated in the negative regulation of several pathways, in particular the JAK/STAT pathway, which can in turn induce SOCS expression and form a negative feedback circuit. The transcriptional upregulation of SOCS-3 confirms that the functional overexpression of STAT3-MER induces the activation of the classical LIF-dependent negative feedback mechanism (for a review see [39]). Previously Duval et al. (2000) showed that expression of SOCS-3, but not SOCS-1 and SOCS-2, was stimulated in ES cells in presence of LIF. The author further demonstrated that, uncontrolled overexpression of SOCS-3 leads to repression of LIF-dependent transcription and severely reduces cell viability. This suggests that the disturbance of a well-balanced SOCS protein content has adverse effects on cell survival [40]. Since the FVB ES cells overexpressing STAT3-MER were viable and pluripotent, it is safe to assume that the SOCS-3 upregulation observed in presence of OHT is a modulatory reaction due to the overproduction of STAT3 in these cells. Through this compensatory mechanism the cells are able to maintain a properly activated LIF signalling cascade. It seems that the upregulation of SOCS-3 is a direct transcriptional activation mediated through STAT3 because the promoters of both mouse and rat SOCS-3 genes contain putative STAT1/STAT3 binding elements, which are necessary and sufficient for LIF-dependent activation of the SOCS-3 promoter activity in reporter assays [41,42].

We also found protein phosphatase 1 regulatory subunit 15B (PP1rs15B) and the elongation initiation factor 2 subunit 2 (eIF2s2) to be among the upregulated genes. PP1rs15B is a constitutively expressed inhibitory subunit of PP1, one of the major eukaryotic serine/threonine phosphatases. PP1rs15B dephosphorylates the α-subunit of eIF2 [43]. The microarray data confirm an upregulation of both PP1rs15B and eIF2s2 indicating that these proteins may be involved in the control of the STAT3 overexpression, whether these genes have a function in maintenance of pluripotency is still unclear.

In the second group we found hexokinase II to be increased in cells overexpressing STAT3, this confirms the importance of this isozyme for embryo viability and indicates that a correct energetic balance is extremely important in the late stages of preimplantation and at the beginning of postimplantation in the embryos.

The third group of differentially expressed genes comprises molecules involved in the maintenance of pluripotency and cell viability. Some of the identified genes were previously correlated with pluripotency [44] or with embryo viability in somatic nuclei derived cloned blastocysts [45].

Lefty2 is regulated by pathways such as Smad2/3 and WNT and by OCT-3/4, which support stemness. Lefty is also induced upon exit from the state of stemness, including forced in vitro differentiation and LIF withdrawal. When LIF is withdrawn, the expression of Lefty increases within 48 hours of cytokine withdrawal [37]. Similarly, retinoic acid that induces differentiation leads to increased expression of Lefty in mouse embryonic carcinoma cells [46]. Differentiation of stem cells to embryoid bodies also leads to increased expression of Lefty in vitro [47]. Therefore, Lefty might be important both to the stemness and differentiation events that follow the exit from this state.

Murine Pem/Rhox5 is an X-linked homeobox-containing gene [48,44], whose homeodomain shares important structural features with two other homeobox genes, which are expressed in extra embryonic lineages and during spermatogenesis [49,50]. The Pem/Rhox5 protein is expressed in the late morula stage, in TE and ICM of blastocyst and after implantation in extra embryonic tissues, in the parietal and visceral endoderm, but not in the primitive ectoderm derivatives. Pem/Rhox5 is also expressed in ES cells, in primordial germ cells and in teratocarcinoma cell lines [51]. Overexpression of Pem/Rhox5 had no phenotype in ES cells, but completely inhibited differentiation into the three primary cell lineages, when ES cells were cultured as embryoid bodies in suspension without LIF [52]. Two different models of action for Pem/Rhox5 are possible. Fan et al. (1999) suggested that Pem/Rhox5 first helps to maintain the undifferentiated cell state, and in a second step promotes a defined cell population of undifferentiated stem cells for differentiation into extra embryonic lineages [52]. Sasaki et al (1991) proposed an alternative in which Pem/Rhox5 directs early differentiation to specific lineages, but does maintain actively the undifferentiated state [44]. Our data are in line with previous studies and indicate that Pem/Rhox5 plays an important role in maintaining pluripotency of ES cells in absence of LIF (Figure 4)

Furthermore, overexpression of STAT3-MER induced differential expression of four genes (Dppa3, NDP52l1, Pramel6, Pramel7) that were identified as a set of OCT-3/4 related genes that were not correctly reactivated in somatic nuclei derived cloned embryos and therefore represent genes that are necessary for embryo viability [45]. Dppa3 (developmental pluripotency-associated 3; Stella; PGC7; Crg1) is preferentially expressed in primordial germ cells, oocytes and preimplantation embryos. In blastocysts, Dppa3 is expressed in TE and ICM and in the early postimplantation embryos Dppa3 expression disappears. The expression re-emerges when at day E7.5 the first primordial germ cells (PGCs) appear [53]. Dppa3 knockout mice are compromised in development; some embryos develop to the two or four cell stage, but fail to reach 8-cell stage [45,54]. Dppa3 was proposed by Sato et al. to play a role in germ line specification in mice by preventing nascent germ cell populations from a somatic cell fate and by retaining their pluripotency [53]. The embryonic function of NDP52l1 (Nuclear dot protein 52) is to date unclear but it is capable of forming dimers and contains leucine zipper motifs indicating a possible function in splicing processes [55].

Pramel6 and Pramel7 (preferentially expressed antigen in melanoma like 6 and 7) are prevalently expressed in preimplantation embryos and embryonic pluripotent cells [45]. Our results confirm these expression patterns and clearly show that whereas Pramel6 is typically expressed in all cells of the morula and blastocyst, Pramel7 is expressed only in the inner part of the morula and in the ICM of the blastocyst. The function of the Pramel genes in embryonic development is unknown, but interestingly, PRAME inhibits retinoic-acid induced differentiation in mouse embryonic carcinoma F9 cells [56]. Recently Kaji et al (2006) showed that Pramel6 and Pramel7 expression is mediated by Mbd3, a component of the nucleosome remodelling and histone deacetylation (NuRD) complex [57]. Kaji et al. proposed that the Mbd3/NuRD-mediated silencing of Pramel6 and Pramel7 in ES cells offers an epigenetic environment in which Mbd3/NuRD is not absolutely required but facilitates differentiation. Furthermore the authors describe that Mbd3 deficiency leads to down regulation of Dppa3 in ES cells. Taken altogether, expression pattern analysis suggests that Dppa3, Pramel6 and Pramel7 are collaborating in deciding the fate of ES cells.

We were further interested in clarifying if some of the identified STAT3-pathway related genes could individually maintaining pluripotency in ES cells. Pem/Rhox5 and Pramel7 were chosen for this experiments: Pramel7 because of its characteristic expression pattern in the central part of the morula and in the inner cell mass of the blastocyst, an expression pattern which is very similar to the one observed for Nanog [25] and is typical for a gene involved in maintenance of pluripotency. Pem/Rhox5 because it was previously described in the literature as a gene blocking the differentiation capacity of the ICM and of ES cells [52,51]. Overexpression of Pramel7 and Pem/Rhox5 in ES cells and cultivation in absence of LIF, allowed the maintenance of pluripotency, as shown by IHC with the typical pluripotency related markers OCT-3/4 and SSEA-1 and with alkaline phosphatase staining, indicating the importance of these proteins in maintaining ES cells in a pluripotent state (Figure 4).

## Conclusion

In summary the data presented here indicates that the overexpression of functional STAT3-MER in FVB/N blastocysts sustains and facilitates the establishment of germline competent ES cells in absence of LIF. Our findings open up the possibility of establishing germline competent ES cells from non-permissive mouse strains by manipulation of the STAT3 signal transduction pathway. Furthermore, gene expression analysis of these transgenic cells cultivated in presence of OHT showed that 26 genes were differentially expressed compared to WT cells cultivated in presence of LIF. By *in situ *hybridization analysis it was possible to identify four up-regulated genes (Hexokinase II, Lefty2, Pramel7, PP1rs15B) whose expression was restricted to the ICM of the blastocysts. Overexpression of two of the upregulated genes, Pem/Rhox5 and Pramel7, in E14 cells and cultivation in absence of LIF demonstrated that these two genes are able to maintain the embryonic stem cells in a pluripotent state without addition of LIF to the culture media.

## Methods

### Generation, Identification, and Maintenance of Transgenic Mice

The pCAGmusstat3ER plasmid containing the full length sequence of murine STAT3 cDNA fused to the ligand-binding domain of mouse estrogen receptor under the control of the chicken β-actin promoter [15] was propagated in *Escherichia coli *DH5α and the minigene was excised with NotI. The fragments were purified from a 1% agarose gel with a Qiaquick extraction kit (Qiagen, Basel, Switzerland) and processed as described. Nuclear injections into fertilized FVB/N oocytes were carried out by conventional methods [27,28]. Transgenic founder were identified by PCR using the β-actin promoter specific primer ggbactfor2: (5'-GGG TTC GGC TTC TGG CGT G-3') and a STAT3 specific primer mmSTAT3back2: (5'-CCA AGG TGC CAG GAA CTG CCG-3'). Two primers specific for the TAG-1 gene (TAG82B: 5'-ACA CGA AGT GAC GCC CAT CCG T-3'; TAG83F: 5'-GGA GGA GAG AGA CCC CGT GAA A-3') were used as a positive control for both wild-type and transgenic mice. ggbactfor2 and mmSTAT3back2 generate a 397 bp band whereas TAG82B and TAG83F generate a 300 bp product.

### Cell culture

#### ES Cell Medium for FVB/N ES cells

KSR-KDMEM (Invitrogen) with 1000 units per ml human LIF, KSR-KDMEM w/o LIF and KSR-KDMEM w/o LIF with 1 μM 4-hydroxytamoxifen (OHT, Sigma).

#### ES Cell Medium for E14 ES cells

G-MEM (Sigma) containing 100 mM sodium pyruvate (GIBCO), 10%FBS, 50 mM β-mercaptoethanol and containing 10^7 ^U/ml ESGRO murine LIF (Chemikon Int.).

#### Embryonic feeder fibroblasts (MEF)

were derived from explanted day 14 foetuses of CD-1-M-TKneo strain mice [29]. Prior to co-culturing with ES cells, confluent layers of MEF (passage 2) cells were treated with mitomycin C (10 μg/μl) for 2.5 hrs and extensively washed. Growth arrested fibroblasts were used as feeder cells for up to one week.

### Embryo recovery, embryo culture, isolation of ES cell lines

Donor females of strains FVB/N (RCC Füllinsdorf, Switzerland and Harlan Horst Postbus, Netherlands) were induced to ovulate by an intra peritoneal injection of 5 I.U. of PMSG (Pregnant mare serum gonadotropin, Folligon, Intervet), followed 46 h later by an intra peritoneal injection of 5 I.U. HCG (human chorionic gonadotropin: Chorulon, Intervet). Subsequently, donor females were mated with STAT3-MER transgenic male mice. Morulae were flushed from the uterotubal junction 3 days after mating and cultured overnight in M16 medium (Sigma). Fully expanded blastocysts were transferred onto MEF in KSR-KDMEM, KSR-KDMEM without LIF with 1 μM OHT or KSR-KDMEM without LIF and cultured at 37°C in an atmosphere of 10 % CO_2 _in air for 6–7 days without media changes. Full-grown ICMs were picked from the outgrown TE and transferred into droplets of trypsin-EDTA solution (Invitrogen) by a mouth-controlled glass-capillary. The ICMs were dissociated mechanically into groups of cells and these aggregates reseeded onto embryonic feeder fibroblasts. 3–4 days later, compact stem cell colonies could be identified. Single colonies were dissociated as described above and reseeded. Subsequently, non-differentiating clonal lines were partly frozen, partly passaged onto 6 cm plates, and split after 2–3 generations for further characterization.

### Karyotype and Sex Determination of ES cell lines

For chromosome counts, ES cells were pre-treated for 3 hrs with colcemide (Sigma, 0.05 μg/ml) and metaphase spreads were prepared according to Triman et al. (1975). Sex determination was carried out using primers specific for the smcy (Selected Mouse cDNA on Y) a gene that maps to the short arm of the mouse Y-chromosome and its X-chromosome homolog smcx (Selected Mouse cDNA on X). The primers bind to both homologues but amplify fragments of different sizes on the X and the Y chromosome. Female cell lines exhibit a single band of 341 bp whereas male cell lines exhibit 2 bands of 341 and 312 bp respectively. The following primers were used: SMC4-1: 5'-CTG AAG CCT TTG GCT TTG AGC AAG CTA C-3'; SMCX-1: 5'-CAA AGA ATT TGG CAG CGG TTT CCC T-3'.

### Generation of injection chimeras

C57BL/6 host embryos were recovered at the morula stage from the oviducts of hormonally treated females and cultured overnight (see above). Blastocysts were transferred into drops of M16 medium (Sigma) and ES cells into drops of HEPES-buffered ES-medium. About 10–15 ES-cells were injected into each blastocyst. After a recovery period of about 2 hrs, injected blastocysts were transferred into the uterine horns of pseudo pregnant NMRI foster mothers (Harlan, England). Chimeric offspring were identified by the absence of coat color pigmentation. Chimeric males were set up to breed at the age of about 8 weeks.

### Western Blotting

ES cell cultures were homogenized with RIPA buffer (50 mM Tris-Cl pH 7.4, 1% NP-40, 0.25% Sodium Deoxycholate, 150 mM NaCl). Protein concentration was determined with BCA-Method (Pierce). Samples were subjected to SDS-PAGE and blotted onto PVDF membranes (Millipore, Volketswil, Switzerland) at 100 V for 1–2 h at 4°C. Immunodetection and chemiluminescent visualization were performed as recommended by the supplier of the chemiluminescence blotting kit (Roche Diagnostics, Rotkreuz, Switzerland). Anti STAT3 antibody (C-20) was purchase from Santa Cruz Biotechnology, anti-phospho (Y705) was purchased from New England Biolabs.

### Immunohistochemistry and alkaline phosphatase staining

For alkaline phosphatase staining the cells were washed with CMF-PBS and fixed in 4% paraformaldehyde in PBS. The cells were washed with PBT (PBS with 0.1% triton X) and incubated in alkaline phosphatase buffer (100 mM Tris-Cl pH9.5, 50 mM MgCl_2_, 100 mM NaCl) containing the AP-substrates nitrotetrazolium-blue and X-phosphate (Roche Diagnostics, Rotkreuz, Switzerland).

For immunohistochemistry the cells were washed in PBS and fixed with 4% paraformaldehyde in PBS. Cells were washed with PBT and incubated with the primary antibodies diluted in PBT containing 2% (v/v) horse serum. Secondary fluorescence labelled antibodies were used for detection. Anti OCT-3/4 (N-19) antibodies were purchased by Santa Cruz Biotechnology, SSEA-1 (Mouse mAb) by Chemicon International and Alexa Fluor 488 anti mouse and Alexa Fluor 594 anti rabbit secondary antibodies were purchased from Molecular Probes.

### cRNA labelling and Hybridization of microarrays

#### cDNA synthesis 1^st ^round of amplification

RNA was combined with 1 μg of T7-dT primer (5'-GGC CAG TGA ATT GTA ATA CGA CTC ACT ATA GGG AGG CGG-(dT)24-3') in a total volume of 12 μl and incubated at 70°C for 10 min. The reaction was placed on ice and the following reagents added in a total volume of 20 μl: first strand buffer (1×), DTT (10 mM), dNTP mix (500 μM), and Superscript III (200 units, Invitrogen) and incubated at 42°C for 2 h. Second strand synthesis was performed by adding the following reagents in a total volume of 150 μl: second strand buffer (1×), dNTP mix (200 μM), *E. coli *DNA ligase (10 units), *E. coli *DNA polymerase (40 units), RNase H (2 units), and incubated at 16°C for 2 h. T4 DNA polymerase (10 units) was then added to fill in the ends of the cDNA and incubated at 16°C for an additional 15 min. Following phenol:chloroform extraction and ethanol precipitation, the cDNA was resuspended in RNase-free water. Transcription was performed using Ambion's (Austin, USA) MEGAscript reagents. A 20 μl reaction containing cDNA, NTP mix (7.5 mM), reaction buffer (1×), and 2 μl enzyme mix was incubated at 37°C for 4 h and the primary cRNA was purified using the RNeasy kit (Qiagen, Chatsworth, CA) per the manufacturer's specifications. Two hundred nanograms of the purified cRNA were then carried forward in the secondary amplification. Samples with less than 200 ng cRNA were concentrated to a 5 μl volume and the entire sample used for the next round of amplification.

#### cDNA synthesis 2^nd ^round of amplification

First-strand cDNA was synthesized by incubating the above cRNA with 1 μg of random primers (Invitrogen, Carlsbad, CA) at 70°C for 10 min. The reaction was then placed on ice and the following reagents added in a 20-μL reaction: first-strand buffer (1×), DTT (10 mM), dNTP mix (500 μM), and Superscript III (200 units). The reaction was incubated at 42°C for 2 h. RNase H (2 units) was added to the reaction and incubated at 37°C for 20 min and then heat-inactivated. The cDNA was combined with 1 μg of the T7-dT primer and incubated at 70°C for 10 min. The reaction was then placed on ice and the following reagents added for a final volume of 150 μl: second-strand buffer (1×), dNTP mix (200 μM), *E. coli *DNA Polymerase (40 units), and then incubated at 16°C for 2 h. T4 DNA polymerase (10 units) was added to fill in the ends of the cDNA and incubated at 16°C for an additional 15 min. The cDNA was purified with phenol:chloroform and precipitated with ethanol.

#### Biotin secondary IVT

The secondary transcription reaction was performed using the Enzo BioArray HighYield RNA kit (Affymetrix, Santa Clara, CA). The secondary cDNA was incubated at 37°C for 4 h in a 40 μl reaction as indicated by in the manufacturer recommendations and the labelled cRNA purified using the RNeasy kit.

### Array Hybridizations and Analysis

The hybridizations were performed with the Murine Genome U74v2 Set, consisting of three GeneChip^® ^probe arrays (U74Av2, U74Bv2 and U74Cv2), together they contain probe sets corresponding to approximately 36,000 full-length mouse genes and EST clusters from the UniGene database. The biotin-labeled cRNA was fragmented in 40 mM Tris-acetate buffer pH 8.1 containing 100 mM potassium acetate and 30 mM Magnesium acetate. 15 μg of every labelled cRNA was mixed with the appropriate buffers and hybridized to respectively a mouse U74Av2, U74Bv2 and U74Cv2 (Affymetrix) for 16 h at 45°C. Computer analysis of the resulting data was performed using the dCHIP software package. Samples were run on triplicate microarrays (three independently cultivated, isolated and labelled WT probes and three independently cultivated, isolated and labelled STAT3-MER probes) and the resulting data combined into subsets and compared using dCHIP. The 18 .CEL files generated by the Affymetrix Microarray Suite (MAS) were converted in .DCP files using dCHIP. The .DCP files were normalized and raw gene expression data generated. Comparison was performed using the dCHIP software wherein the three WT FVB ES cells were designated as "baseline" (B) and the three STAT3-MER overexpressing ES cells designated as "experiment" (E). Genes expressed 1.5 fold higher or lower in the WT versus STA3-MER cells were then identified by defining the appropriate filtering criteria in the dCHIP software (E/B < 1.5 or B/E > 1.5; E-B > 100 or B-E > 100, P < 0.1, t-test). Calculations were performed with both the PM/MM (perfect match/mismatch) difference model and the PM (perfect match) only model in order to compare the results. The microarray data is deposited in the Gene Omnibus Repository (GEO), accession number GSE11398.

### Real-Time Quantitative PCR (Q-PCR)

The total RNA from cultured ES cells was obtained using Qiagen RNeasy mini-kit and reverse transcribed with oligo-dT primers (Invitrogen) and SuperscriptIII (Invitrogen). Quantitative Real time experiments were performed with the SyberGreen technology using the QuantiTect SYBR kit (Qiagen) and a Rotorgene 6000 cycler (Corbett). For quantitation of gene expression comparative Ct-method was used after normalization with β-actin. The following primers were used:

βactin_fwd: 5'-cat cca ggc tgt gct gtc cct gta tgc-3'

βactin_bwd: 5'-gat ctt cat ggt gct agg agc cag agc-3'

Lefty2_fwd: 5'-aca gcg cgg atg tgg agg aga tgg-3'

Lefty2_bwd: 5'-atc ctc acg gac tct cag cca ttc a-3'

Eif2s2_fwd: 5'-tac atc gtc aac cca aac atc tcc ttg c-3'

Eif2s2_bwd: 5'-ggc acg gag ctg tgc tcg ctt-3'

Dppa3_fwd: 5'-agg gtc cgc act ttg ttg tcg gtg c-3'

Dppa3_bwd: 5'-gct cct aat tct tcc cga ttt tcg cat-3'

Hexokinase2_fwd: 5'-tgt ggt ggc cgt ggt aaa tg-3'

Hexokinase2_bwd: 5'-tct tga ggc gct ctg aga tg-3'

NDP5211_fwd: 5'-cat gag cag cta cag agg aag ca-3'

NDP5211_bwd: 5'-gtg cct cag att cac tgt gta gct aa-3'

Nanog_fwd: 5'-aca agg gtc tgc tac tga gat gc-3'

Nanog_bwd: 5'-gga gac ttc ttg cat ctg ctg g-3'

Pem/Rhox5_fwd: 5'-ctt ccg tgg aca aga gga ag-3'

Pem/Rhox5_bwd: 5'-tgt cat agc cgg cat atg tg-3'

Ppp1r15b_fwd: 5'-gcc ttc aag ctg gtc tag tc-3'

Ppp1r15b_bwd: 5'-cat cgc tat caa agc cat cg-3'

Pramel6_fwd: 5'-cag gaa gac gag tgg caa agc acg t-3'

Pramel6_bwd: 5'-agc cct gga atc tca tag cct aca tc-3'

Pramel7_fwd: 5'-gag gag aag cag aac atc agc aag a-3'

Pramel7_bwd: 5'-ctc tta gag gcg tga cat cta ggt t-3'

Stat3_fwd: 5'-ggc aag ggc ttc tcc ttc tg-3'

Stat3_bwd: 5'-agc tgc tgc ttg ttg gtg tat gg-3'

Skp2_fwd: 5'-cag ctg ctc cag act gga tg-3'

Skp2_bwd: 5'-ggt tcc ctc tgg cac gat tc-3'

Socs3_fwd: 5'-cct cca gca tct ttg tcg gaa gac-3'

Socs3_bwd: 5'-tac tga tcc agg aac tcc cga atg-3'

Trim41_fwd: 5'-tga gcc gca tgt ttt gtc agg ctg c-3'

Trim41_bwd: 5'-c aca ctt cgc gct gga cta gga gct-3'

Quantitative RT-PCR for each gene was done in triplicates and the values were normalized to the corresponding amounts of β-actin RNAs.

### In situ hybridization

Templates for riboprobe-synthesis were obtained by amplification through RT-PCR. Briefly, total RNA was isolated from FVB/STAT3-MER ES cells cultivated in presence of 4OHT and RT was performed with oligo-dT primers. 300–400 bp long fragments containing part of the ORF of the genes of interest were amplified by PCR and cloned in a pCRII^® ^TOPO^® ^dual promoter vector (Invitrogen). DNA templates for riboprobe synthesis were digested with appropriate enzymes to provide fragments for either sense or antisense orientation of the PCR product respect to the vector. Labelled riboprobes were synthesized by SP6 or T7 RNA polymerases by incorporation of digoxygenin-labelled UTPs.

The embryos were transferred in a micro pore insert (12 μm) sitting in a well of a 4 well plate and fixed with freshly prepared 4% PFA/PBS, washed twice in PBT and dehydrated once in 25%, 50%, 75% and twice in 100% methanol/PBT. The dehydration was followed by rehydration in the reverse order of the MeOH/PBT series 75%, 50%, 25% for 5 min each. The embryos permeabilized in RIPA buffer and refixed in 4% PFA/0.2% glutaraldehyde. After prehybridisation in hybridisation solution for ≥2 hours at 70°C the embryos were incubated in hybridisation solution containing 1.6 μg/ml of the corresponding riboprobe. As a control for the specificity of the labelling, in each hybridization experiment control embryos were hybridized with an equal concentration of a sense probe transcribed from the same template as the antisense probe. After high and low stringency washes hybridized riboprobes were detected using an AP-coupled anti-digoxygenin antibodies (Roche Diagnostics, Rotkreuz, Switzerland) and the AP-substrate BM-Purple (Roche Diagnostics, Rotkreuz, Switzerland). The staining reaction was stopped by rinsing in 2 mM EDTA/PBT. Embryos were subsequently post-fixed in 4% PFA/0.1% glutaraldehyde in PBT and cleared in a glycerol:PBT 1:1 solution.

### Overexpression of Pramel7, Pem/Rhox5 and Nanog

The full length open reading frames (ORF) of Pramel7 and Pem/Rhox5 were amplified by RT-PCR and sequenced. The full length cDNAs were cloned in the pfloxedNanog expression vector [25] by exchanging the Nanog cDNA with either the one of Pem/Rhox5 or Pramel7. The expression vectors were completely sequenced and finally electroporated in E14 ES cells. After selection with puromycin single colonies were isolated and their expression levels were determined by real time PCR. The clones with the strongest overexpression were chosen for further experiments.

## Abbreviations

ES cell: embryonic stem cell; ICM: inner cell mass; LIF: Leukemia Inhibitory factor: MEF: mouse embryonic fibroblasts; OCT-3/4: octamer-binding transcription factor 3/4; OHT: 4-Hydroxytamoxyfen; PRAME: preferentially expressed in melanoma; RTQ-PCR: reverse transcription quantitative PCR; STAT3: signal transducer and activator of transcription 3; STAT3-MER: STAT3 fusioned with a modified estrogen receptor; TE: trophectoderm.

## Authors' contributions

PC coordinated the project, conducted the experiments, analyzed the data and drafted the manuscript. EAC, SU and PL participated in performing experiments and acquisition and interpretation of data. TY and TM contributed in preparation of expression vectors. TR generated the transgenic mice by pronuclear injection. BL established the ES cell lines and performed the blastocyst injections. KB participated in coordination of the study and helped to draft the manuscript. All authors read and approved the final manuscript.

## References

[B1] Evans MJ, Kaufman MH (1981). Establishment in culture of pluripotential cells from mouse embryos. Nature.

[B2] Ledermann B (1997). Establishment of embryonic stem cell lines. Immunological Methods Manual.

[B3] Martin GR (1981). Isolation of a pluripotent cell line from early mouse embryos cultured in medium conditioned by teratocarcinoma stem cells. Proc Natl Acad Sci U S A.

[B4] Smith AG, Hooper ML (1987). Buffalo rat liver cells produce a diffusible activity which inhibits the differentiation of murine embryonal carcinoma and embryonic stem cells. Dev Biol.

[B5] Williams RL, Hilton DJ, Pease S, Willson TA, Stewart CL, Gearing DP, Wagner EF, Metcalf D, Nicola NA, Gough NM (1988). Myeloid leukaemia inhibitory factor maintains the developmental potential of embryonic stem cells. Nature.

[B6] Pesce M, Scholer HR (2001). Oct-4: gatekeeper in the beginnings of mammalian development. Stem Cells.

[B7] Rossant J (2001). Stem cells from the Mammalian blastocyst. Stem Cells.

[B8] Kishimoto T, Taga T, Akira S (1994). Cytokine signal transduction. Cell.

[B9] Taga T, Kishimoto T (1997). Gp130 and the interleukin-6 family of cytokines. Annu Rev Immunol.

[B10] Burdon T, Smith A, Savatier P (2002). Signalling, cell cycle and pluripotency in embryonic stem cells. Trends Cell Biol.

[B11] Stahl N, Farruggella TJ, Boulton TG, Zhong Z, Darnell JE, Yancopoulos GD (1995). Choice of STATs and other substrates specified by modular tyrosine-based motifs in cytokine receptors. Science.

[B12] Fukada T, Hibi M, Yamanaka Y, Takahashi-Tezuka M, Fujitani Y, Yamaguchi T, Nakajima K, Hirano T (1996). Two signals are necessary for cell proliferation induced by a cytokine receptor gp130: involvement of STAT3 in anti-apoptosis. Immunity.

[B13] Dudley DT, Pang L, Decker SJ, Bridges AJ, Saltiel AR (1995). A synthetic inhibitor of the mitogen-activated protein kinase cascade. Proc Natl Acad Sci U S A.

[B14] Burdon T, Stracey C, Chambers I, Nichols J, Smith A (1999). Suppression of SHP-2 and ERK signalling promotes self-renewal of mouse embryonic stem cells. Dev Biol.

[B15] Matsuda T, Nakamura T, Nakao K, Arai T, Katsuki M, Heike T, Yokota T (1999). STAT3 activation is sufficient to maintain an undifferentiated state of mouse embryonic stem cells. Embo J.

[B16] Stewart CL, Kaspar P, Brunet LJ, Bhatt H, Gadi I, Kontgen F, Abbondanzo SJ (1992). Blastocyst implantation depends on maternal expression of leukaemia inhibitory factor. Nature.

[B17] Li M, Sendtner M, Smith A (1995). Essential function of LIF receptor in motor neurons. Nature.

[B18] Ware CB, Horowitz MC, Renshaw BR, Hunt JS, Liggitt D, Koblar SA, Gliniak BC, McKenna HJ, Papayannopoulou T, Thoma B (1995). Targeted disruption of the low-affinity leukemia inhibitory factor receptor gene causes placental, skeletal, neural and metabolic defects and results in perinatal death. Development.

[B19] Yoshida K, Taga T, Saito M, Suematsu S, Kumanogoh A, Tanaka T, Fujiwara H, Hirata M, Yamagami T, Nakahata T, Hirabayashi T, Yoneda Y, Tanaka K, Wang WZ, Mori C, Shiota K, Yoshida N, Kishimoto T (1996). Targeted disruption of gp130, a common signal transducer for the interleukin 6 family of cytokines, leads to myocardial and hematological disorders. Proc Natl Acad Sci U S A.

[B20] Nichols J, Chambers I, Taga T, Smith A (2001). Physiological rationale for responsiveness of mouse embryonic stem cells to gp130 cytokines. Development.

[B21] Raz R, Lee CK, Cannizzaro LA, d'Eustachio P, Levy DE (1999). Essential role of STAT3 for embryonic stem cell pluripotency. Proc Natl Acad Sci U S A.

[B22] Kuhn R, Rajewsky K, Muller W (1991). Generation and analysis of interleukin-4 deficient mice. Science.

[B23] Taketo M, Schroeder AC, Mobraaten LE, Gunning KB, Hanten G, Fox RR, Roderick TH, Stewart CL, Lilly F, Hansen CT (1991). FVB/N: an inbred mouse strain preferable for transgenic analyses. Proc Natl Acad Sci U S A.

[B24] Schoonjans L, Kreemers V, Danloy S, Moreadith RW, Laroche Y, Collen D (2003). Improved generation of germline-competent embryonic stem cell lines from inbred mouse strains. Stem Cells.

[B25] Chambers I, Colby D, Robertson M, Nichols J, Lee S, Tweedie S, Smith A (2003). Functional expression cloning of Nanog, a pluripotency sustaining factor in embryonic stem cells. Cell.

[B26] Mitsui K, Tokuzawa Y, Itoh H, Segawa K, Murakami M, Takahashi K, Maruyama M, Maeda M, Yamanaka S (2003). The homeoprotein Nanog is required for maintenance of pluripotency in mouse epiblast and ES cells. Cell.

[B27] Littlewood TD, Hancock DC, Danielian PS, Parker MG, Evan GI (1995). A modified oestrogen receptor ligand-binding domain as an improved switch for the regulation of heterologous proteins. Nucleic Acids Res.

[B28] Akira S, Nishio Y, Inoue M, Wang XJ, Wei S, Matsusaka T, Yoshida K, Sudo T, Naruto M, Kishimoto T (1994). Molecular cloning of APRF, a novel IFN-stimulated gene factor 3 p91-related transcription factor involved in the gp130-mediated signaling pathway. Cell.

[B29] Becker S, Groner B, Muller CW (1998). Three-dimensional structure of the Stat3beta homodimer bound to DNA. Nature.

[B30] Li C, Hung Wong W (2001). Model-based analysis of oligonucleotide arrays: model validation, design issues and standard error application. Genome Biol.

[B31] Chambers I, Silva J, Colby D, Nichols J, Nijmeijer B, Robertson M, Vrana J, Jones K, Grotewold L, Smith A (2007). Nanog safeguards pluripotency and mediates germline development. Nature.

[B32] Sekkai D, Gruel G, Herry M, Moucadel V, Constantinescu SN, Albagli O, Tronik-Le Roux D, Vainchenker W, Bennaceur-Griscelli A (2005). Microarray analysis of LIF/Stat3 transcriptional targets in embryonic stem cells. Stem Cells.

[B33] Sharov AA, Piao Y, Matoba R, Dudekula DB, Qian Y, VanBuren V, Falco G, Martin PR, Stagg CA, Bassey UC, Wang Y, Carter MG, Hamatani T, Aiba K, Akutsu H, Sharova L, Tanaka TS, Kimber WL, Yoshikawa T, Jaradat SA, Pantano S, Nagaraja R, Boheler KR, Taub D, Hodes RJ, Longo DL, Schlessinger D, Keller J, Klotz E, Kelsoe G, Umezawa A, Vescovi AL, Rossant J, Kunath T, Hogan BL, Curci A, D'Urso M, Kelso J, Hide W, Ko MS (2003). Transcriptome analysis of mouse stem cells and early embryos. PLoS Biol.

[B34] Ramalho-Santos M, Yoon S, Matsuzaki Y, Mulligan RC, Melton DA (2002). "Stemness": transcriptional profiling of embryonic and adult stem cells. Science.

[B35] Ivanova NB, Dimos JT, Schaniel C, Hackney JA, Moore KA, Lemischka IR (2002). A stem cell molecular signature. Science.

[B36] Larsen L, Ropke C (2002). Suppressors of cytokine signalling: SOCS. Apmis.

[B37] Duval D, Reinhardt B, Kedinger C, Boeuf H (2000). Role of suppressors of cytokine signaling (Socs) in leukemia inhibitory factor (LIF) -dependent embryonic stem cell survival. Faseb J.

[B38] Auernhammer CJ, Bousquet C, Melmed S (1999). Autoregulation of pituitary corticotroph SOCS-3 expression: characterization of the murine SOCS-3 promoter. Proc Natl Acad Sci U S A.

[B39] Paul C, Seiliez I, Thissen JP, Le Cam A (2000). Regulation of expression of the rat SOCS-3 gene in hepatocytes by growth hormone, interleukin-6 and glucocorticoids mRNA analysis and promoter characterization. Eur J Biochem.

[B40] Jousse C, Oyadomari S, Novoa I, Lu P, Zhang Y, Harding HP, Ron D (2003). Inhibition of a constitutive translation initiation factor 2alpha phosphatase, CReP, promotes survival of stressed cells. J Cell Biol.

[B41] Sasaki AW, Doskow J, MacLeod CL, Rogers MB, Gudas LJ, Wilkinson MF (1991). The oncofetal gene Pem encodes a homeodomain and is regulated in primordial and pre-muscle stem cells. Mech Dev.

[B42] Bortvin A, Eggan K, Skaletsky H, Akutsu H, Berry DL, Yanagimachi R, Page DC, Jaenisch R (2003). Incomplete reactivation of Oct4-related genes in mouse embryos cloned from somatic nuclei. Development.

[B43] Oulad-Abdelghani M, Chazaud C, Bouillet P, Mattei MG, Dolle P, Chambon P (1998). Stra3/lefty, a retinoic acid-inducible novel member of the transforming growth factor-beta superfamily. Int J Dev Biol.

[B44] Dvash T, Mayshar Y, Darr H, McElhaney M, Barker D, Yanuka O, Kotkow KJ, Rubin LL, Benvenisty N, Eiges R (2004). Temporal gene expression during differentiation of human embryonic stem cells and embryoid bodies. Hum Reprod.

[B45] Maiti S, Doskow J, Sutton K, Nhim RP, Lawlor DA, Levan K, Lindsey JS, Wilkinson MF (1996). The Pem homeobox gene: rapid evolution of the homeodomain, X chromosomal localization, and expression in reproductive tissue. Genomics.

[B46] Han YJ, Park AR, Sung DY, Chun JY (1998). Psx, a novel murine homeobox gene expressed in placenta. Gene.

[B47] Li Y, Lemaire P, Behringer RR (1997). Esx1, a novel X chromosome-linked homeobox gene expressed in mouse extraembryonic tissues and male germ cells. Dev Biol.

[B48] Lin TP, Labosky PA, Grabel LB, Kozak CA, Pitman JL, Kleeman J, MacLeod CL (1994). The Pem homeobox gene is X-linked and exclusively expressed in extraembryonic tissues during early murine development. Dev Biol.

[B49] Fan Y, Melhem MF, Chaillet JR (1999). Forced expression of the homeobox-containing gene Pem blocks differentiation of embryonic stem cells. Dev Biol.

[B50] Sato M, Kimura T, Kurokawa K, Fujita Y, Abe K, Masuhara M, Yasunaga T, Ryo A, Yamamoto M, Nakano T (2002). Identification of PGC7, a new gene expressed specifically in preimplantation embryos and germ cells. Mech Dev.

[B51] Payer B, Saitou M, Barton SC, Thresher R, Dixon JP, Zahn D, Colledge WH, Carlton MB, Nakano T, Surani MA (2003). Stella is a maternal effect gene required for normal early development in mice. Curr Biol.

[B52] Sadler I, Crawford AW, Michelsen JW, Beckerle MC (1992). Zyxin and cCRP: two interactive LIM domain proteins associated with the cytoskeleton. J Cell Biol.

[B53] Epping MT, Wang L, Edel MJ, Carlee L, Hernandez M, Bernards R (2005). The human tumor antigen PRAME is a dominant repressor of retinoic acid receptor signaling. Cell.

[B54] Kaji K, Caballero IM, MacLeod R, Nichols J, Wilson VA, Hendrich B (2006). The NuRD component Mbd3 is required for pluripotency of embryonic stem cells. Nat Cell Biol.

[B55] Brinster RL, Chen HY, Trumbauer ME, Yagle MK, Palmiter RD (1985). Factors affecting the efficiency of introducing foreign DNA into mice by microinjecting eggs. Proc Natl Acad Sci U S A.

[B56] Wilmut I, Hooper ML, Simons JP (1991). Genetic manipulation of mammals and its application in reproductive biology. J Reprod Fertil.

[B57] Stewart CL, Schuetze S, Vanek M, Wagner EF (1987). Expression of retroviral vectors in transgenic mice obtained by embryo infection. Embo J.

